# A Temperature-Robust Non-Enzymatic Lactate Sensor Based on Graphene Fiber Composite Electrodes

**DOI:** 10.3390/nano16161032

**Published:** 2026-08-20

**Authors:** Qianqian Zhong, Feng Han, Weixuan Jing, Yifan Zhao, Kun Zheng, Song Wang, Yaxin Zhang, Dejiang Lu, Chenying Wang, Binbin Jiao, Zhuangde Jiang

**Affiliations:** 1 School of Instrument Science and Technology, Xi’an Jiaotong University, Xi’an 710049, China; zhongqianqian@stu.xjtu.edu.cn (Q.Z.);; 2 State Key Laboratory for Manufacturing Systems Engineering, Xi’an Jiaotong University, Xi’an 710049, China; 3 International Joint Laboratory for Micro/Nano Manufacturing and Measurement Technologies, Xi’an Jiaotong University, Xi’an 710049, China

**Keywords:** non-enzymatic lactate sensor, graphene fiber, electrochemical deposition, wide-temperature stability, wearable devices

## Abstract

Real-time lactate monitoring is essential for clinical diagnostics, sports physiology, and industrial bioprocessing, yet conventional enzymatic sensors suffer from limited stability, narrow operational temperature range, and complex fabrication protocols. Herein, we report a robust non-enzymatic electrochemical sensor based on graphene fibers (GFs), featuring a GF/Au/Ni(OH)_2_ composite electrode with controllable structure fabricated via sequential electrodeposition. Systematic optimization of deposition parameters established a quantitative relationship between surface architecture and electrochemical response, revealing a critical trade-off between active site density and charge transport efficiency. The sensor achieved optimal performance when both Au and Ni(OH)_2_ were deposited for 900 s, exhibiting a high sensitivity of 1.24 mA mM^−1^ cm^−2^ and a remarkably broad operational temperature range of 0–100 °C. Moreover, the sensor demonstrates excellent repeatability, superior anti-interference capability against common electroactive species, and outstanding long-term durability with 97.8% response retention after 14 days. This work provides a rational design strategy for balancing catalytic activity and transport properties in metal–metal oxide composites, offering a reliable platform for advanced applications in next-generation wearable health-monitoring systems.

## 1. Introduction

Real-time monitoring of lactate concentration is of paramount importance in clinical diagnostics, sports physiology, and industrial bioprocessing, as abnormal lactate levels serve as critical biomarkers for conditions such as tissue hypoxia, sepsis, and metabolic disorders [1,2,3]. Traditional lactate detection relies predominantly on enzyme-based electrochemical sensors, which, despite their high specificity, suffer from inherent drawbacks including the limited stability of enzymes, susceptibility to environmental factors (pH, temperature, humidity), and complex immobilization protocols [4,5,6]. To overcome these limitations, non-enzymatic electrochemical sensors have emerged, leveraging robust inorganic catalytic materials to achieve significantly enhanced operational stability and substantially expanded applicability [7,8,9].

Among various catalytic materials, transition metal hydroxides, particularly Ni(OH)_2_, garnered considerable attention due to their excellent electrocatalytic activity toward lactate oxidation in alkaline media. The catalytic mechanism involves the reversible redox transition between Ni(OH)_2_ and NiOOH, where the higher-valence NiOOH species mediates the dehydrogenation of lactate to pyruvate [8,10,11]. However, the intrinsically low electrical conductivity of Ni(OH)_2_ impedes electron percolation from the catalytic layer to the underlying conductive scaffold, thereby limiting the rate at which electrons reach interfacial active sites and participate in the Ni^2+^/Ni^3+^ redox transformation and subsequent lactate oxidation [12,13]. To circumvent this issue, hybrid architectures incorporating conductive nanostructures have been proposed, such as those based on gold nanoparticles (AuNPs) [14,15] and platinum nanoparticles (PtNPs) [16]. In these architectures, the metallic component facilitates rapid electron transport while the metal hydroxide provides abundant active sites [17,18]. The resulting metal–metal oxide (MMO) heterostructures exhibit synergistic effects that significantly enhance electrocatalytic performance [19,20]. Nevertheless, the rational design of such composite electrodes remains challenging, particularly with respect to controlling interfacial morphology and optimizing component loading to balance catalytic activity with mass transport efficiency [21,22]. Recent efforts have explored diverse MMO-based configurations for lactate detection, including NiO nanoparticles (24 μA mM^−1^ cm^−2^) [10], NiS hollow nanostructures (0.65 μA mM^−1^ cm^−2^, limit of detection (LOD) 0.5 μM) [23], Ni(OH)_2_-modified ITO electrodes (132.50 μA mM^−1^ cm^−2^, LOD 6.7 μM) [24], and NiOOH/Ni(OH)_2_ core–shell architectures (80.00 μA mM^−1^ cm^−2^, LOD 0.15 μM) [25]. Active site density has been effectively modulated through nanomorphology engineering; however, these systems generally exhibit limited sensitivity and are confined to room-temperature operation on rigid substrates. The simultaneous achievement of high sensitivity, wide temperature adaptability, and mechanical flexibility remains elusive.

Fiber-shaped microelectrodes have emerged as a promising platform for next-generation wearable sensors, offering distinct advantages including high aspect ratio, mechanical flexibility, and enhanced current densities [26,27,28]. GFs are especially attractive as substrates due to their outstanding electrical conductivity, large specific surface area, and excellent mechanical strength. The hierarchically wrinkled structure of GFs, arising from volumetric contraction during thermal reduction, not only imparts favorable flexibility but also significantly enhances the specific surface area, providing ample active sites for subsequent functionalization [29,30,31]. The wrinkled architecture enables uniform deposition of secondary phases, which makes GFs ideal scaffolds for constructing MMO-based composite electrodes [10,25,32]. Despite these merits, systematic studies that correlate the deposition parameters of MMO components with the resultant surface morphology and electrochemical performance remain scarce, hindering the rational design of high-performance fiber-based sensors.

In this work, a GF/Au/Ni(OH)_2_ composite fiber electrode with a well-controlled architecture was fabricated through sequential electrodeposition by successively depositing Au and Ni(OH)_2_ onto graphene fibers. The synergistic combination of the superior conductivity of Au and the catalytic activity of Ni(OH)_2_ yields a high-performance non-enzymatic lactate sensor. By systematically tuning the electrodeposition times, we established a quantitative relationship among process parameters, surface morphology, and electrochemical response, which revealed a trade-off between active site density and charge and mass transport efficiency. The optimal sensor was obtained by depositing 900 s on both components, which demonstrated an outstanding sensitivity of 1.24 mA mM^−1^ cm^−2^ and stable performance within a wide temperature range (0 to 100 °C), thus meeting various operational requirements from low-temperature transportation to sterilization processes. Beyond sensitivity, the device delivered highly reproducible signals, maintained performance in complex media, and retained stability over prolonged periods, collectively validating its viability for incorporation into wearable and implantable health-monitoring systems. This study not only provides a facile strategy for fabricating flexible non-enzymatic sensors but also offers fundamental insights into the morphology–performance correlation in MMO-based composite electrodes.

## 2. Experimental Section

### 2.1. Materials and Reagents

GO fibers were formed by drying a 12 mg mL^−1^ aqueous dispersion in a polytetrafluoroethylene (PTFE) mold at 180 °C, and then reduced in Ar (99.999%) at 900 °C to obtain graphene fibers. Chloroauric acid (HAuCl_4_·4H_2_O, Au > 47.8%) was used for subsequent electrochemical modification. Anhydrous ethanol (CH_3_CH_2_OH) was a product of Tianjin Fuyu Fine Chemical Co., Ltd. (Tianjin, China) and was used for washing and terminating the reaction.

### 2.2. Fabrication of the GF/Au/Ni(OH)_2_ Non-Enzymatic Electrochemical Lactate Sensor

A single GF functioned as the working electrode, while a platinum wire and an Ag/AgCl electrode served as the counter and reference electrodes, respectively. Au particles and Ni(OH)_2_ nanoparticles were sequentially electrodeposited onto the GF surface using a CHI 660E electrochemical workstation. The Au deposition solution was 0.01 M HAuCl_4_ (Sinopharm Chemical Reagent Co., Ltd., Shanghai, China, Au content > 47.8%). The electrodeposition was carried out at a constant potential of −0.2 V for 500–1100 s to obtain GF/Au with different Au loadings. Subsequently, the specimens were submerged in a 10 mM Ni(NO_3_)_2_·6H_2_O solution (Sigma-Aldrich St. Louis, MO, USA) and subjected to electrodeposition at −0.9 V for 300–1200 s to form the GF/Au/Ni(OH)_2_ heterointerface. After deposition, the specimens were gently rinsed three times with ultrapure water and then vacuum-dried at 25 °C for 12 h, yielding a set of enzyme-free lactate sensors.

### 2.3. Characterization and Measurements

The microstructure of graphene fibers was examined with an SU-8010 field-emission SEM (Hitachi, Ltd., Tokyo, Japan). The HR800 laser Raman spectrometer (Horiba Jobin Yvon, Paris, France) provided Raman spectral data, while the ESCALAB Xi+ X ray photoelectron spectrometer (Thermo Fisher, Waltham, MA, USA) enabled analysis of surface chemistry and electronic structure. All voltammetric and impedance tests were run on a CHI 660E potentiostat (Shanghai Chenhua Co., Ltd., Shanghai, China).

## 3. Results and Discussion

**Figure 1:**
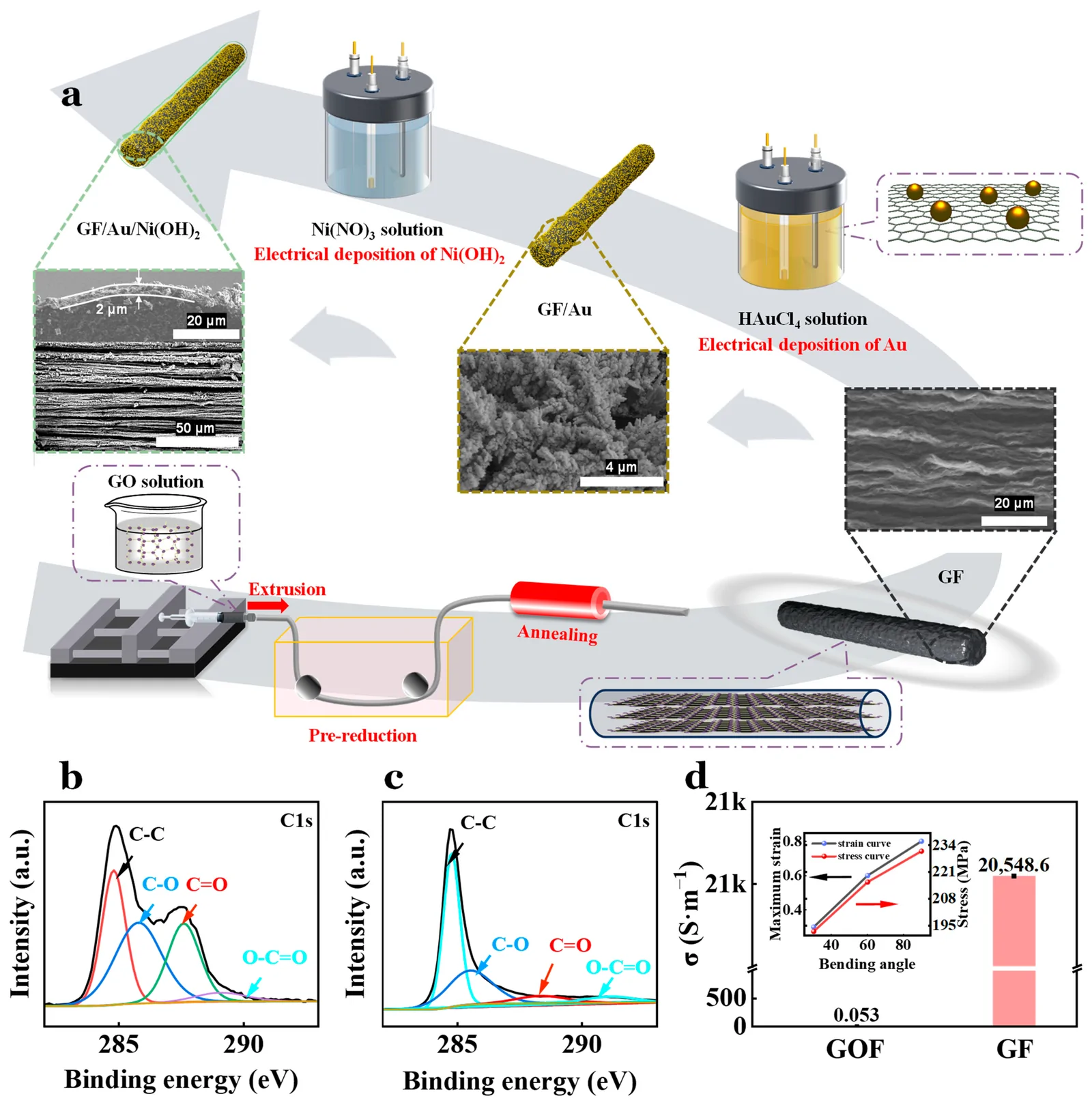
Fabrication and fundamental characterization of the GF/Au/Ni(OH)_2_ composite fiber electrode. (**a**) A schematic diagram of the preparation process of the GF/Au/Ni(OH)_2_ non-enzymatic lactate sensor, including the extrusion of GO solution, pre-reduction, thermal annealing, and sequential electrode deposition of Au and Ni(OH)_2_, and typical SEM images of GF, GF/Au, and GF/Au/Ni(OH)_2_ are embedded. The upper left illustration shows that the thickness of the catalytic layer is within 2 μm to demonstrate the evolution process of the surface morphology. (**b**,**c**) High-resolution C 1s XPS spectra of (**b**) GOF and (**c**) GF, demonstrating the effective removal of oxygen-containing functional groups upon thermal annealing. (**d**) Electrical conductivity comparison between GOF and GF, with the inset showing the maximum strain and stress curves of graphene fibers under varying bending angles.

The fabrication of the GF/Au/Ni(OH)_2_ composite fiber electrode follows a three-step protocol comprising wet-spinning, thermal annealing, and sequential electrodeposition, as schematically illustrated in Figure 1a. A 12 mg mL^−1^ aqueous GO dispersion was then injected into a PTFE mold with an inner diameter of 2 mm and dried at 180 °C to form GO fibers. The extruded fibers underwent thermal reduction in Ar atmosphere (99.999%) at 900 °C to yield GFs with restored sp^2^ conjugated networks. The resulting fibers exhibited a uniform cylindrical geometry with an average diameter of approximately 130 μm, originating from the shrinkage of the extruded GO dispersion within the PTFE mold. For electrochemical modification, a single GF served as the working electrode, with a platinum wire and an Ag/AgCl electrode functioning as the counter and reference electrodes, respectively. Au nanoparticles were electrodeposited from a 0.01 M HAuCl_4_ solution at a constant potential of −0.2 V for 500–1100 s, followed by Ni(OH)_2_ deposition from a 10 mM Ni(NO_3_)_2_·6H_2_O solution at −0.9 V for 300–1200 s. After deposition, the specimens were gently rinsed with ultrapure water and vacuum-dried at 25 °C for 12 h.

The surface morphological evolution of the fiber electrodes at each fabrication stage was systematically examined by field-emission scanning electron microscopy (FE-SEM), with representative micrographs embedded within Figure 1a. The pristine GF exhibits a highly wrinkled surface texture, which arises from volumetric contraction during solvent evaporation and high-temperature thermal reduction. During this process, the GO sheets within the fibers experience irregular stacking driven by capillary forces and van der Waals interactions, generating numerous surface wrinkles from the nano- to micro-scale. Such a wrinkled architecture not only imparts favorable flexibility and mechanical robustness to the fibers but also significantly enhances their specific surface area, thereby providing ample active sites for subsequent functionalization [33,34]. Upon Au electrodeposition, the fiber surface undergoes substantial morphological transformation, with the formation of distinctive “leaf-like” nanostructures uniformly distributed across the graphene surface. This indicates robust interfacial bonding between the Au nanoparticles and the underlying graphene substrate, and the hierarchical architecture significantly boosts the fiber’s surface-to-volume ratio while establishing an optimal support matrix for Ni(OH)_2_ attachment. Following sequential deposition of Ni(OH)_2_, the nanoparticles uniformly coat both the graphene fiber surface and the Au particle surfaces, forming a dense catalytic layer approximately 2 μm in thickness. The resulting hierarchical porous architecture of the GF/Au/Ni(OH)_2_ composite further enlarges the solid–liquid interfacial area, offering a strong structural basis for the enhanced electrochemical performance of the non-enzymatic lactate sensor.

The chemical transformation from graphene oxide fiber (GOF) to GF during thermal annealing was further corroborated by X-ray photoelectron spectroscopy (XPS). Deconvolution of the C 1s region for the synthesized GOF (Figure 1b) reveals four characteristic components assigned to C–C (284.8 eV), C–O (285.8 eV), C=O (287.6 eV), and O–C=O (289.2 eV), confirming the presence of numerous oxygen-containing functional groups on the oxidized fiber surface. After thermal annealing at 900 °C, the GF (Figure 1c) exhibits a substantial decrease in both the intensity and relative content of these oxygenated peaks, with a pronounced decline in C=O bonds and relatively limited alterations in C–O and O–C=O bonds. The atomic carbon-to-oxygen (C/O) ratio increased significantly from 2.65 for GOF to 22.80 for GF, demonstrating that high-temperature annealing effectively reduces graphene oxide. This elevated C/O ratio reflects both the recovered conjugated architecture within the graphene fibers and the reduced density of oxygen-containing groups, attesting to an enhanced degree of reduction.

The charge transport properties of the fiber electrodes were evaluated by electrical conductivity measurements, as summarized in Figure 1d. The GOF exhibits remarkably low electrical conductivity of merely 0.053 S m^−1^, which originates from extensive surface oxygen functionalities that generate sp^3^ hybridization defects and compromise the conjugated π-electron architecture. After annealing at 900 °C, the electrical conductivity of the obtained GF sample dramatically increases to 20,548.6 S m^−1^, representing an enhancement of over three orders of magnitude. This trend is fully consistent with the variation in C/O ratio measured by XPS: high-temperature annealing thermally removes oxygen-containing functional groups in graphene oxide, promotes the reconstruction of the sp^2^ conjugated network, and thereby increases both carrier concentration and mobility. The inset of Figure 1d presents the mechanical characterization of graphene fibers under flexural loading, showing the variations in maximum strain and maximum stress with bending angle. As the imposed displacement on both sides of the graphene fiber electrode increases, the bending radius of curvature decreases, leading to a continuous rise in both maximum strain and maximum stress. At a bending angle of 90°, the maximum stress concentrated in the inner central region of the bend reaches 231 MPa. Based on the intrinsic material properties of graphene fibers, the maximum stress should remain below 400 MPa and the number of bending cycles should not exceed 1000 to prevent fiber fracture. As a base electrode, the graphene fiber therefore possesses excellent electrical conductivity while maintaining stable conductivity under bending conditions, fulfilling the fundamental requirements for incorporation into flexible and wearable sensing platforms.

**Figure 2:**
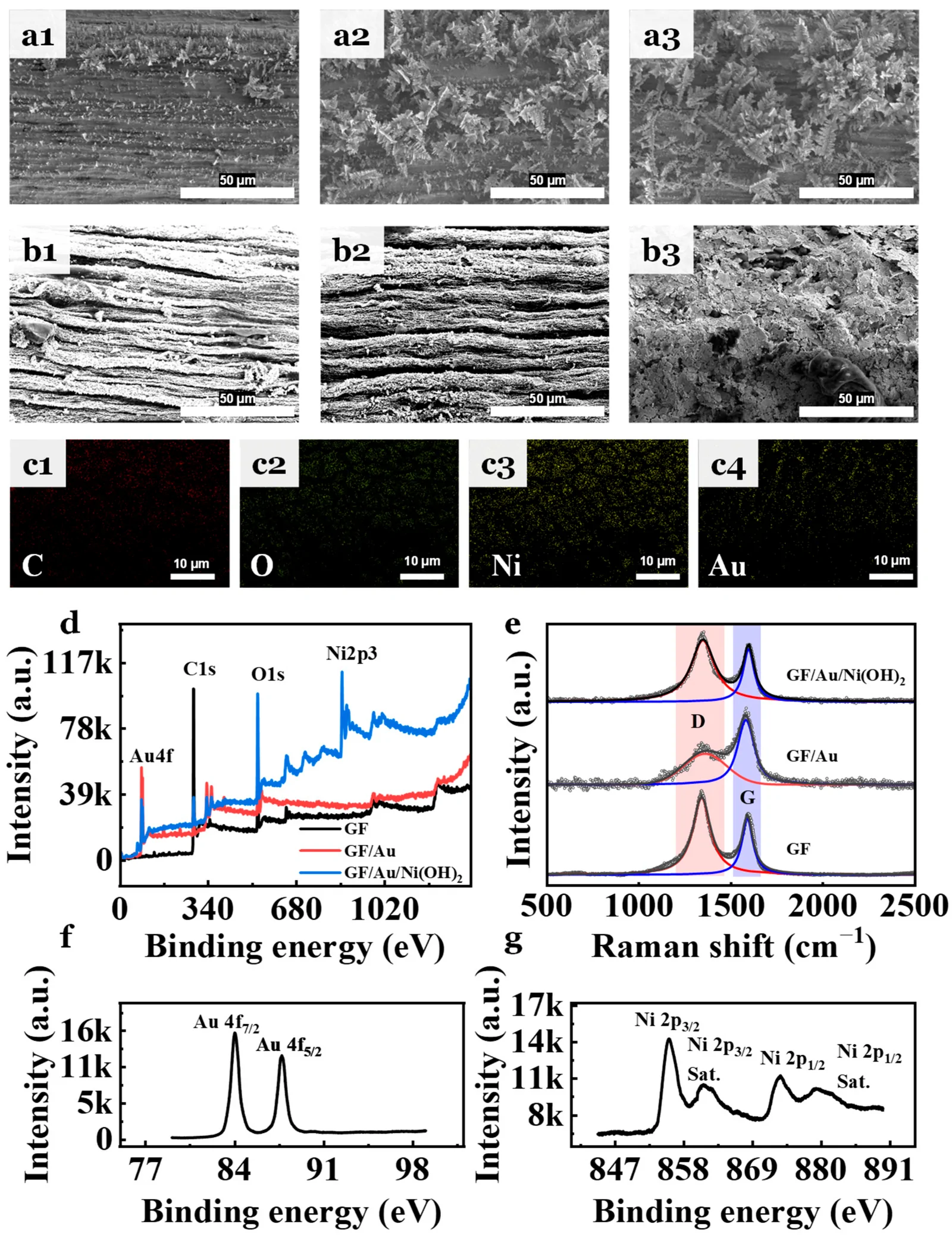
Morphological and spectroscopic characterization of GF/Au and GF/Au/Ni(OH)_2_ composite electrodes. (**a1**–**a3**) SEM images of GF/Au electrodes with Au deposition times of 500, 700, and 1100 s, showing the progressive densification of leaf-like Au nanostructures. (**b1**–**b3**) SEM images of GF/Au/Ni(OH)_2_ electrodes with Ni(OH)_2_ deposition times of 300, 600, and 1200 s, illustrating the evolution from isolated nanoparticles to a continuous catalytic layer. (**c1**–**c4**) EDS elemental mapping images of the GF/Au/Ni(OH)_2_ composite electrode, displaying the spatial distributions of C, O, Ni, and Au, respectively. The Ni signal distributes uniformly across the fiber surface with localized intensity variations, indicating a semi-continuous Ni(OH)_2_ layer, while the partially detectable Au signal and attenuated C signal confirm that the catalytic coating does not achieve complete encapsulation of the underlying conductive scaffold. (**d**) XPS survey spectra of GF, GF/Au, and GF/Au/Ni(OH)_2_ composite fibers. (**e**) Raman spectra of GF, GF/Au, and GF/Au/Ni(OH)_2_, with peak fitting for the D and G bands. (**f**,**g**) High-resolution XPS spectra of (**f**) Au 4f and (**g**) Ni 2p regions for the GF/Au/Ni(OH)_2_ composite, confirming the elemental states of Au and Ni(OH)_2_.

The surface architecture of the composite electrodes was systematically modulated by tuning the electrodeposition parameters, and the resultant morphological evolution was characterized by FE-SEM. For Au deposition, preliminary tests spanning 100 to 1500 s identified three regimes analogous to Shu et al.’s dendrite-like gold nanostructure system where time-dependent morphological evolution follows nucleation–adsorption–growth–branching [35]. Figure 2(a1–a3) present the SEM images of GF/Au electrodes prepared with Au deposition times of 500, 700, and 1100 s, respectively. At 500 s, the graphene fiber surface is sparsely decorated with Au nanoparticles that partially retain the underlying wrinkled texture of the GF substrate. As the deposition time extends to 700 s, the density of Au particles increases markedly, with the leaf-like nanostructures becoming more prominent and interconnected. At 1100 s, the Au layer achieves nearly complete surface coverage, forming a densely packed nanostructured film that substantially obscures the original graphene morphology. This progressive densification of Au nanostructures with prolonged deposition time significantly augments the solid–liquid interfacial contact area and furnishes an enhanced conductive scaffold for subsequent Ni(OH)_2_ loading. For Ni(OH)_2_, screening over 100 to 1800 s revealed a progression from discrete nanoparticles to continuous films analogous to Guan et al.’s Cu_2_O@Cu/TiO_2_ system [36], with 300, 600, and 1200 s capturing nucleation-dominated, growth-dominated, and coalescence-dominated regimes, respectively. Figure 2(b1–b3) display the surface morphology of GF/Au/Ni(OH)_2_ electrodes fabricated with Ni(OH)_2_ deposition times of 300, 600, and 1200 s, respectively, while maintaining the optimal Au deposition condition. At 300 s, Ni(OH)_2_ appears as isolated nanoparticles dispersed across the GF/Au surface, leaving substantial exposed regions of the underlying Au nanostructures. Upon extending the deposition to 600 s, the Ni(OH)_2_ coverage becomes more uniform, with nanoparticles aggregating into a semi-continuous layer that begins to envelop the Au leaf-like protrusions. At 1200 s, the Ni(OH)_2_ layer evolves into a thick, continuous coating that fully blankets the composite surface, effectively burying the Au nanostructures beneath. This structural evolution from discrete nanoparticles to a dense catalytic film underscores the critical role of deposition duration in governing the surface coverage and thickness of the Ni(OH)_2_ layer. The chemical composition and electronic structure of the GF, GF/Au, and GF/Au/Ni(OH)_2_ electrodes were investigated by XPS. Figure 2d presents the survey spectra of the three samples. The pristine GF exhibits dominant C 1s (~284.8 eV) and O 1s (~532.3 eV) signals originating from the graphene substrate and surface-adsorbed oxygen-containing functional groups. Upon Au deposition, distinct Au 4f peaks emerge in the GF/Au spectrum, confirming the successful incorporation of Au species. Following Ni(OH)_2_ deposition, the GF/Au/Ni(OH)_2_ spectrum additionally displays a pronounced Ni 2p feature, while the Au 4f signal remains detectable, verifying the coexistence of both metallic and hydroxide components in the composite architecture. High-resolution XPS analysis was performed to elucidate the chemical states of the deposited species. Figure 2f shows the Au 4f spectrum of the GF/Au/Ni(OH)_2_ composite, where spin–orbit doublets are observed at 84.1 eV (Au 4f_7/2_) and 87.5 eV (Au 4f_5/2_). The measured energy separation of 3.4 eV corresponds to the established values for metallic Au, confirming that the electrodeposited Au particles exist in their elemental form on the fiber surface. Figure 2g presents the high-resolution Ni 2p spectrum, exhibiting dominant peaks at 856.0 eV (Ni 2p_3/2_) and 873.6 eV (Ni 2p_1/2_), accompanied by strong satellite peaks that are uniquely characteristic of Ni(OH)_2_. Comparison with standard database values reveals marked deviations from metallic Ni (852.6 eV), NiO (853.7 eV), and NiS (853.1 eV), but strong alignment with Ni(OH)_2_ reference values (855.6 eV). Furthermore, the energy separation between Ni 2p_3/2_ and O 1s (~270.8 eV) provides supplementary verification of Ni(OH)_2_ formation. These XPS results offer definitive evidence, from a chemical-state perspective, for the robust anchoring of Ni(OH)_2_ nanoparticles to the graphene composite fiber. The chemical composition and spatial distribution of the GF/Au/Ni(OH)_2_ composite were investigated by EDS elemental mapping to complement the surface-sensitive XPS analysis. Figure 2(c1–c4) present the spatial distributions of C, O, Ni, and Au across the fiber surface. The C signal localizes primarily to interstitial regions between deposited nanoparticle aggregates, indicating attenuation rather than complete extinction of the graphene substrate signal. The Ni distribution spans the fiber surface with localized intensity variations, revealing a semi-continuous Ni(OH)_2_ layer rather than a perfectly dense film. The O signal correlates closely with Ni distribution, consistent with the hydroxide nature of the catalytic overlayer. Notably, the Au signal remains partially detectable beneath the Ni(OH)_2_ coating, corroborating the Au 4f peaks observed in XPS and confirming that the Au interlayer is not fully buried. This partial coverage architecture, wherein catalytic sites are accessible while the conductive GF/Au backbone remains partially exposed, is advantageous for maintaining electron percolation pathways across the composite electrode.

Raman spectroscopy was employed to probe the graphene lattice structure within the composite fiber electrodes. As depicted in Figure 2e, all three samples display prominent D and G bands near 1360 cm^−1^ and 1580 cm^−1^, which are assigned to defect-induced vibrations of sp^2^-hybridized carbon and in-plane stretching of the graphene lattice, respectively [37,38]. The pristine GF exhibits a D/G band intensity ratio (I_D_/I_G_) of 1.10, reflecting a certain degree of lattice imperfection chiefly ascribed to the material’s porous surface morphology and the residual oxygen-containing functional groups after thermal reduction. In the GF/Au composite, the I_D_/I_G_ ratio decreases to 0.992. This phenomenon is primarily attributed to the surface-enhanced Raman scattering (SERS) effect induced by the deposited Au nanoparticles. The SERS effect preferentially amplifies the G-band intensity due to the strong coupling between the Au surface plasmon resonance and the E_2_g phonon mode of graphene, whereas the D-band—originating from defect-induced double-resonant scattering—exhibits less susceptibility to this enhancement mechanism [39]. The GF/Au/Ni(OH)_2_ hybrid fiber exhibits an upward trend in I_D_/I_G_ ratio after Ni(OH)_2_ deposition. This observation is consistent with the well-established phenomenon that metal and metal hydroxide nanoparticles preferentially nucleate at defect sites on graphene substrates, where the dangling bonds and localized strain fields provide energetically favorable anchoring sites [40,41]. The selective deposition of Ni(OH)_2_ on defect-rich regions enhances the local electromagnetic field and increases the scattering cross-section of the D-band, thereby elevating its relative intensity [42]. Furthermore, the interfacial interaction between Ni(OH)_2_ and graphene introduces additional extrinsic defects at the boundary regions, including lattice distortion and sp^3^ hybridization induced by Ni–O–C coordination bonds [43]. The combined effect of defect-site-selective nucleation, interfacial defect generation, and G-band suppression collectively results in the increased I_D_/I_G_ ratio, reflecting the overall structural disorder of the composite filament rather than an intrinsic increase in graphene defect density.

**Figure 3:**
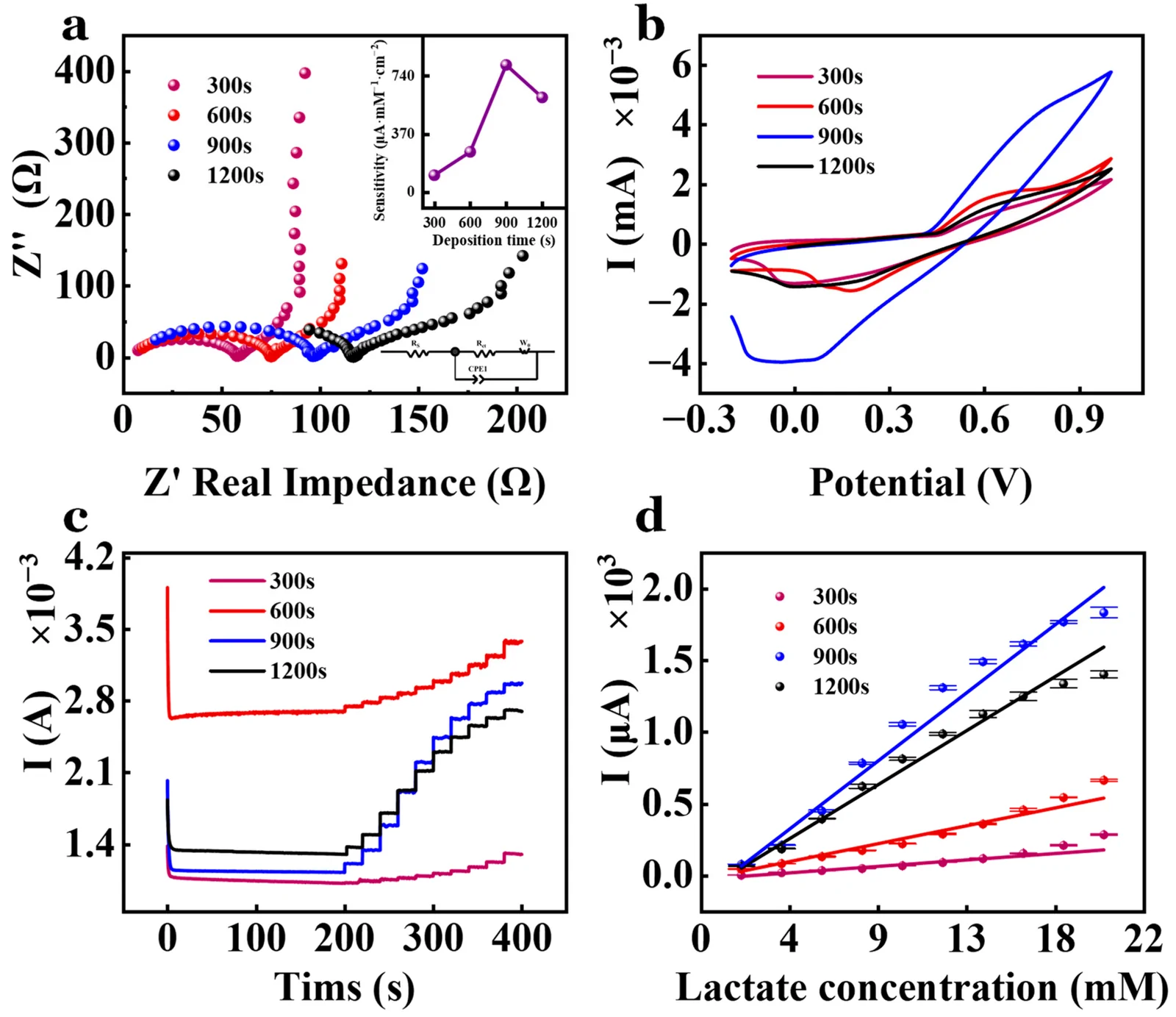
Performance evaluation of GF/Au/Ni(OH)_2_ non-enzymatic lactate sensors with varying Ni(OH)_2_ deposition times. (**a**) EIS Nyquist plots for electrodes with varying Ni(OH)_2_ deposition times (300–1200 s). Inset: EEC employed for qualitative EIS analysis, comprising R_S_, CPE, R_ct_, and W_0_. Right inset: lactate sensitivity as a function of Ni(OH)_2_ deposition time. (**b**) Cyclic voltammetry (CV) curves of the deposited Ni(OH)_2_ lactate sensors in 0.2 M NaOH/0.2 M KCl electrolyte. (**c**) Current-time dynamic response curves of the deposited Ni(OH)_2_ lactate sensors upon successive lactate addition at +0.8 V. (**d**) Linear fitting calibration curves of current–time dynamic response with error bars indicating standard deviation from triplicate measurements.

To isolate the effect of Ni(OH)_2_ loading on the electrocatalytic response, the Ni(OH)_2_ electrodeposition time was systematically varied while the Au underlayer was maintained at a fixed deposition duration. Preliminary SEM imaging indicated that an Au deposition time of 300 s was sufficient to produce a continuous and electrically conductive film across the GF surface, and this duration was therefore selected as the baseline condition. This value is also consistent with standard potentiostatic deposition protocols reported for comparable nickel-based compound electrodeposition on carbon fiber substrates [44]. By holding the Au deposition time constant at this functionally adequate value, the subsequent variation in Ni(OH)_2_ deposition parameters could be evaluated without interference from incomplete or variable electron transfer pathways at the Au-GF interface. Figure 3a presents the electrochemical impedance spectroscopy (EIS) data for electrodes with Ni(OH)_2_ deposition times ranging from 300 to 1200 s. Analysis of the Nyquist plots reveals that longer Ni(OH)_2_ deposition times steadily increase the high-frequency charge transfer resistance, indicating progressively deteriorated interfacial electron transfer kinetics. Concurrently, the low-frequency Warburg region extends with elevated deposition time, reflecting augmented diffusion limitations within the thickened catalytic layer. The equivalent circuit employed for qualitative analysis is shown in the inset, comprising a solution resistance (R_S_) in series with a parallel combination of a constant phase element (CPE), a charge transfer resistance (R_ct_), and a Warburg impedance (W_0_). This trend elucidates a fundamental trade-off between Ni(OH)_2_ loading and electrode performance. While moderate Ni(OH)_2_ deposition provides more catalytic active sites for lactate oxidation, excessive loading engenders thick-film formation or particle stacking that obstructs the underlying graphene conductive network and increases the tortuosity of mass transfer pathways, thereby compromising both electron transport and reactant accessibility [45,46]. In transition-metal-hydroxide-based electrochemical systems, high mass loading without structural optimization typically prevents electrolyte penetration and limits electron transportation to the active surface [46]. The sensitivity peaks at 809.25 μA mM^−1^ cm^−2^ for 900 s deposition, declining to 602.50 μA mM^−1^ cm^−2^ at 1200 s (inset), confirming 900 s as the optimal parameter where active site density and transport efficiency are quantitatively balanced. As shown in Figure 3b, all electrodes exhibit a prominent lactate oxidation peak at approximately 0.65 V, characteristic of the Ni(OH)_2_/NiOOH redox-mediated oxidation mechanism. The oxidation peak current density increases markedly from 300 to 900 s, where maximal catalytic activity is achieved, then decreases at 1200 s. Notably, while the voltammetric responses for 300, 600, and 1200 s retain highly similar curve shapes and anodic peak positions, the 900 s electrode exhibits a distinct shift in the oxidation peak toward higher potentials, accompanied by a steep current rise above 0.6 V. This anomalous behavior at 900 s reflects a critical densification transition wherein prolonged electrodeposition promotes lateral stacking and coalescence of Ni(OH)_2_ nanosheets into a compact layer [47,48]. The resulting increase in charge transfer resistance and restricted OH^−^ diffusion to the interface elevate the overpotential required for the Ni(OH)_2_/NiOOH conversion, thereby shifting the anodic peak to more positive potentials, while the pronounced current increase above 0.6 V stems from an earlier oxygen evolution reaction at edge sites of the densified film. Despite the elevated Rct, the 900 s electrode delivers the highest catalytic response because the amperometric current is governed by the interplay of active site density and interfacial electron transfer efficiency rather than by the latter alone. At 900 s, the film transitions from a sparse island morphology to a densely interconnected catalytic network, maximizing the population of electrochemically accessible Ni(OH)_2_/NiOOH redox centers; the gain in active site density outweighs the kinetic penalty from increased Rct. This trade-off between interfacial conductivity and catalytic site abundance is structurally analogous to composite electrocatalysts where conductive sublayers facilitate electron percolation while surface catalytic layers govern active site availability, as demonstrated in Cu_2_O@Cu/TiO_2_ nanotube arrays where a Cu interlayer reduces R_ct_ by 292-fold while Cu_2_O provides the catalytic sites [36], and in Cu/Cu_2_O/Ni(OH)_2_ composites where rapid electron transfer is achieved through abundant active sites despite the intrinsically poor conductivity of the Cu/Cu_2_O phase [49]. At 1200 s, the film thickness exceeds the effective electron transfer distance, rendering the outer Ni(OH)_2_ layers electrochemically inactive and fixing the effective interfacial environment; consequently, the voltammetric profile reverts to the peak position characteristic of thinner films, while the decreased current reflects the reduced population of accessible active sites. Thus, the 900 s electrode represents the critical balance between maximized active site density and the onset of interfacial transport limitations. Figure 3c displays the current–time dynamic response curves upon successive addition of lactate to the electrolyte. The 900 s electrode exhibits the most pronounced current increment with each lactate concentration step, whereas the 300 s and 600 s counterparts show substantially weaker responses due to insufficient catalytic loading. The 1200 s electrode, despite possessing the highest Ni(OH)_2_ coverage, delivers an intermediate response that is inferior to the 900 s electrode, attributable to the increased charge transfer resistance and diffusion limitations identified in the EIS analysis. Figure 3d presents the corresponding linear calibration curves with error bars representing the standard deviation from triplicate measurements. The 900 s electrode achieves the highest sensitivity of 809.25 μA mM^−1^ cm^−2^ with excellent linearity (R^2^ = 0.98), significantly outperforming the 300 s (108.41 μA mM^−1^ cm^−2^), 600 s (256.71 μA mM^−1^ cm^−2^), and 1200 s (602.50 μA mM^−1^ cm^−2^) counterparts. These results collectively establish 900 s as the optimal Ni(OH)_2_ deposition time, where the synergistic combination of abundant active sites and unimpeded charge transport yields maximal electrocatalytic efficiency.

**Figure 4:**
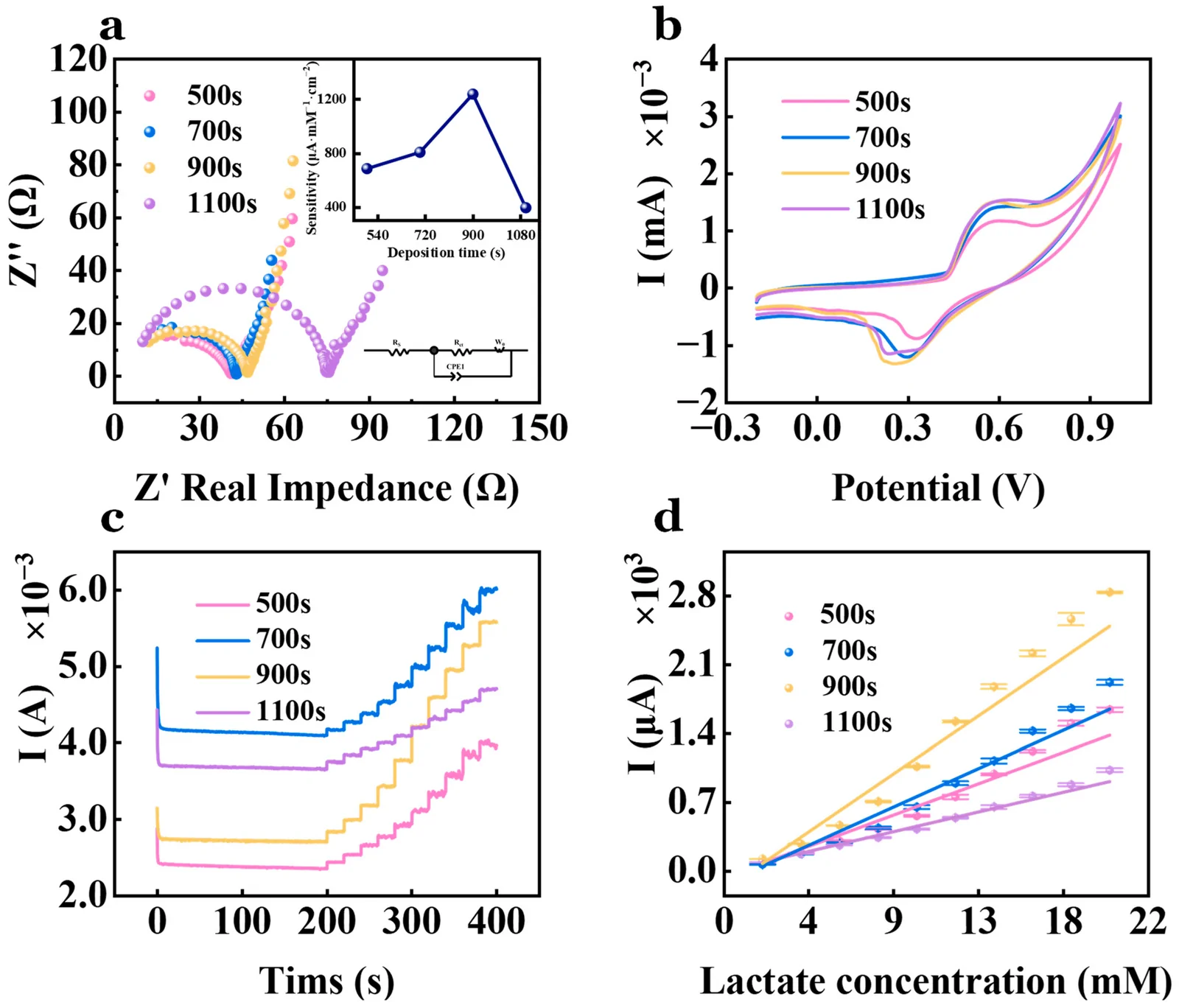
Performance evaluation of GF/Au/Ni(OH)_2_ non-enzymatic lactate sensors with varying Au deposition times (Ni(OH)_2_ deposition time fixed at 900 s). (**a**) EIS Nyquist plots for electrodes with varying Au deposition times (500–1100 s). Inset: EEC employed for qualitative EIS analysis. Right inset: lactate sensitivity as a function of Au deposition time. (**b**) CV curves of the deposited Au lactate sensors in 0.2 M NaOH/0.2 M KCl electrolyte. (**c**) Current–time dynamic response curves of the deposited Au lactate sensors upon successive lactate addition at +0.8 V. (**d**) Linear fitting calibration curves of current–time dynamic response with error bars indicating standard deviation from triplicate measurements.

Building upon the optimal Ni(OH)_2_ deposition time of 900 s, the effect of Au deposition duration was systematically investigated to establish an efficient electron transport network while maintaining catalytic interface accessibility. Figure 4a presents the EIS spectra for electrodes fabricated with Au deposition times ranging from 500 to 1100 s. Analysis of the Nyquist plots indicates that increasing the Au deposition time from 500 to 1100 s progressively elevates the interfacial charge transfer resistance. This phenomenon stems from the interfacial effects of Au nanostructures and the intrinsic insulating nature of Ni(OH)_2_, which collectively impede electron transfer at the electrode–electrolyte interface [50,51]. Nevertheless, electrodes with moderate Au deposition (900 s) maintain efficient charge transfer capability, as reflected by the relatively compact feature in the high-frequency region of the impedance spectrum. The equivalent circuit model is depicted in the inset. The observed evolution in interfacial impedance correlates with the morphological changes revealed by scanning electron microscopy (SEM, Figure 2(a1–a3)), where a pronounced gradient increase in Au nanosheet density on the graphene fiber surface is evident with prolonged electrodeposition time. This morphological evolution not only significantly enhances the specific surface area of the graphene fiber scaffold but also furnishes an optimal support platform for subsequent Ni(OH)_2_ nanoparticle loading. The inset plots the relationship between Au deposition time and lactate sensitivity, revealing a volcano-shaped trend: the sensitivity increases from 685.92 μA mM^−1^ cm^−2^ at 500 s to 807.81 μA mM^−1^ cm^−2^ at 700 s, reaches a maximum of 1239.30 μA mM^−1^ cm^−2^ at 900 s, and subsequently declines to 396.21 μA mM^−1^ cm^−2^ at 1100 s. This optimal performance at 900 s arises from the synergistic balance between the enhanced conductive pathways provided by the Au nanostructures and the unimpeded mass transport through the hierarchically porous architecture. Cyclic voltammetry characterization was conducted to assess the electrocatalytic behavior of the electrodes prepared under varying Au deposition durations. As shown in Figure 4b, all electrodes exhibit similar lactate oxidation profiles, with an onset potential at approximately +0.5 V and a pronounced anodic peak at +0.6 V. Across the 500–900 s deposition window, the peak current demonstrates a progressive rise with extended duration, concomitant with a modest anodic shift in peak position, reflecting enhanced electrode kinetics and improved catalytic turnover. The 900 s electrode displays the largest peak current and the most favorable peak potential, indicative of optimal electrocatalytic activity. The 1100 s electrode, despite possessing the most extensive Au coverage, exhibits a diminished peak current compared to the 900 s counterpart, suggesting that excessive Au loading may block active Ni(OH)_2_ sites or impede reactant diffusion to the catalytic interface. Chronoamperometric measurements were performed at +0.8 V to evaluate the quantitative sensing performance of the GF/Au/Ni(OH)_2_ electrodes. Figure 4c presents the current–time dynamic response curves upon successive addition of lactate. The 900 s electrode demonstrates the most substantial current increment with each concentration step, consistent with its superior sensitivity. The 500 s and 700 s electrodes exhibit progressively weaker responses, attributable to insufficient Au loading and consequently limited conductive scaffold for electron transport. The 1100 s electrode shows an intermediate response that falls below the 900 s electrode, confirming that excessive Au deposition compromises the overall sensing performance despite the increased metallic content. Figure 4d displays the corresponding linear calibration curves with error bars representing the standard deviation from triplicate measurements. The calibration curve for the 900 s electrode (R^2^ = 0.98) achieves a peak sensitivity of 1239.30 μA mM^−1^ cm^−2^, significantly surpassing the 500 s (685.92 μA mM^−1^ cm^−2^), 700 s (807.81 μA mM^−1^ cm^−2^), and 1100 s (396.21 μA mM^−1^ cm^−2^) samples. This confirms 900 s as the optimal Au deposition parameter, where the hierarchical GF/Au/Ni(OH)_2_ architecture maximizes the synergistic coupling between the superior conductivity of Au and the catalytic activity of Ni(OH)_2_.

The superior performance of the optimized GF/Au/Ni(OH)_2_ electrode originates from the rational balance between active site density and charge transport efficiency. The Au nanostructures deposited for 900 s establish an interconnected conductive network that facilitates rapid electron transfer from the Ni(OH)_2_ catalytic sites to the graphene fiber backbone, while the hierarchically wrinkled surface ensures efficient mass transport of lactate molecules and hydroxide ions to the active interface [52,53]. This optimized multi-solid-phase electrode–electrolyte interface promotes reactant accessibility and product desorption, thereby achieving an exceptionally high response current and sensitivity [54].

**Figure 5:**
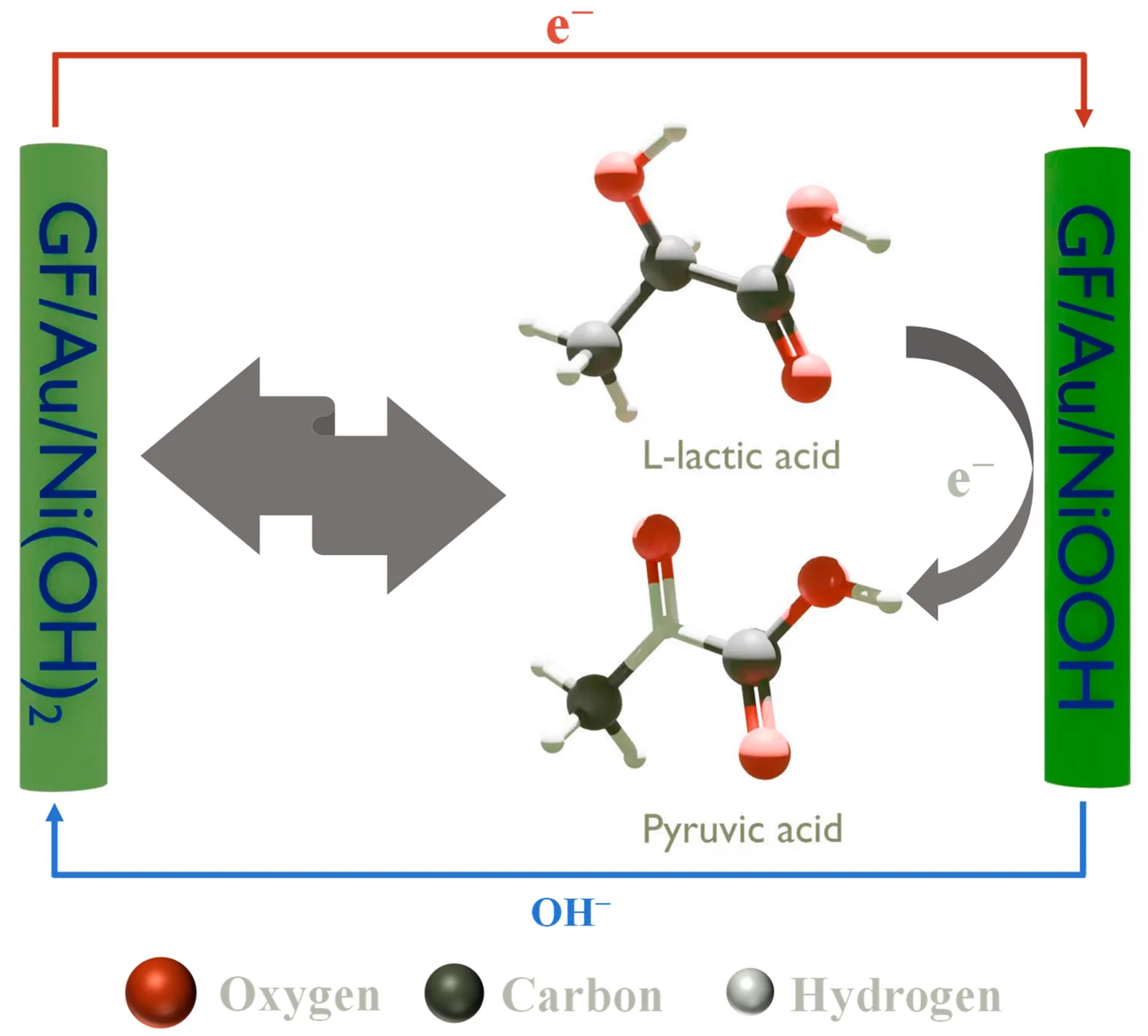
The reaction mechanism of the GF/Au/Ni(OH)_2_ composite fiber working electrode.

The superior architecture of the GF/Au/Ni(OH)_2_ non-enzymatic lactate sensor originates from the Au/Ni(OH)_2_ synergistic coupling [55], which effectively facilitates the rapid migration of electrolyte ions and significantly enhances the kinetics of lactate electrocatalytic oxidation as shown in Figure 5. Lactate molecules (C_3_H_6_O_3_) accumulate on the surface of Ni(OH)_2_ nanoparticles through physical adsorption, where their hydroxyl (-OH) and carboxyl (-COOH) functional groups coordinate with catalytic active sites [11,56,57]. Under the applied electric field, Ni(OH)_2_ is oxidized to the higher-valence NiOOH, simultaneously catalyzing the dehydrogenation of lactate molecules. The resulting pyruvate (C_3_H_4_O_3_) molecules desorb from the catalyst surface, freeing the active sites for subsequent cycles. Concurrently, Ni(OH)_2_ is regenerated via electrochemical reduction, completing the catalytic cycle. This efficient catalytic mechanism benefits from the wrinkled structure of graphene fibers, which provides abundant active sites for the attachment of nanocatalytic materials. Furthermore, the graphene–Au substrate ensures rapid electron transport and increases the contact area at the solid–liquid interface. Lastly, the optimized three phase interface in the GF/Au/Ni(OH)_2_ configuration promotes reactant mass transfer, further enlarging the solid liquid contact area. Electrons generated during the reaction are rapidly transferred through the GF/Au/Ni(OH)_2_ architecture, thereby achieving an exceptionally high response speed. Lactic acid undergoes catalytic oxidation by Au/Ni(OH)_2_ as described by the following equation [10,11,24,25]:

Ni(OH)_2_ + OH^−^ → NiOOH + H_2_O + e^−^ (1)

C_3_H_6_O_3_ + 2NiOOH → C_3_H_4_O_3_ + 2Ni(OH)_2_ (2)

NiOOH + H_2_O + e^−^ → Ni(OH)_2_ + OH^−^ (3)

**Figure 6:**
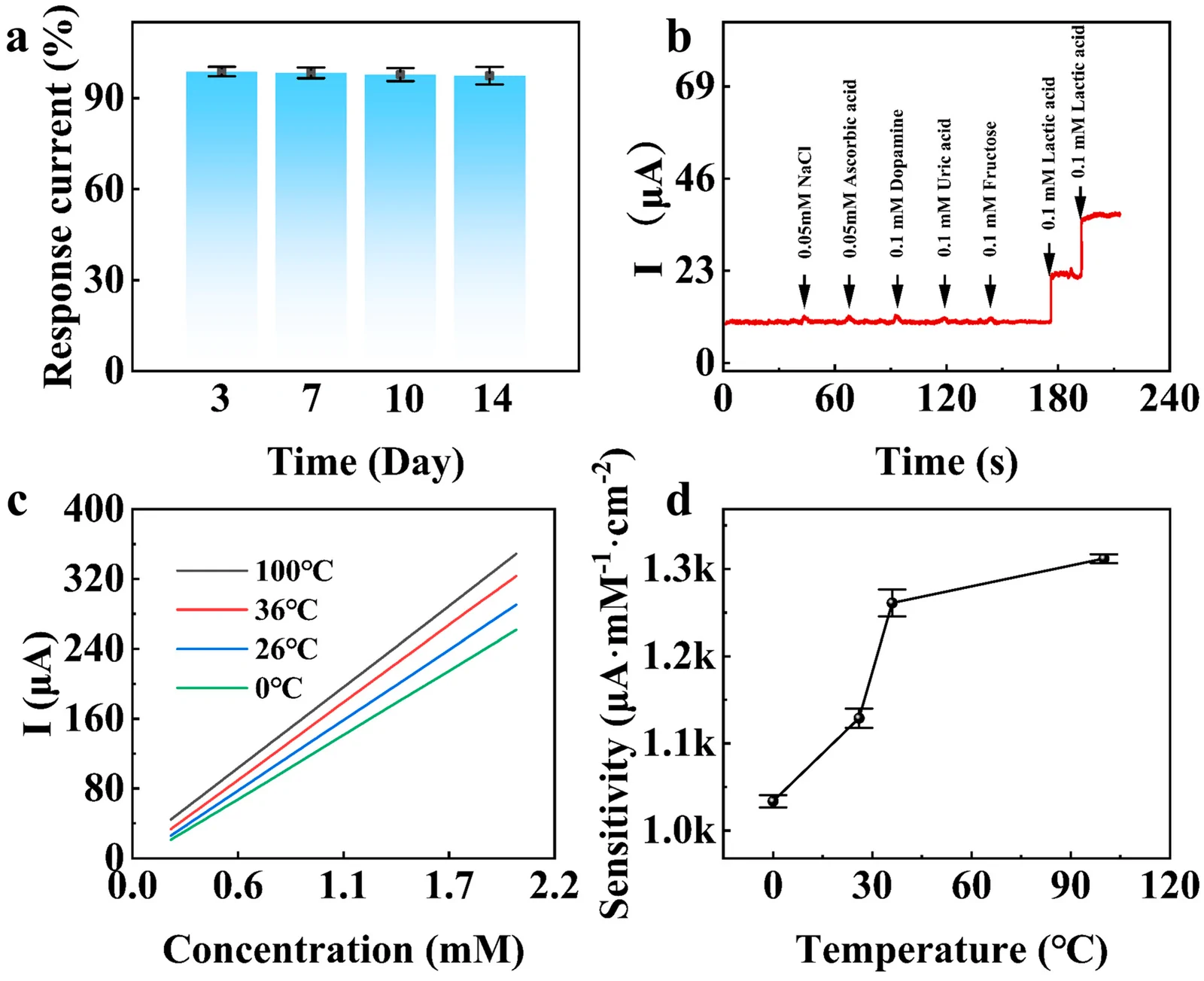
Stability, selectivity, and temperature adaptability of the optimized GF/Au/Ni(OH)_2_ non-enzymatic electrochemical lactate sensor. (**a**) Long-term stability assessment showing the retention of response current to 3 mM lactate over a 14-day period, with error bars indicating standard deviation. (**b**) Anti-interference performance evaluated by sequential addition of 0.05 M NaCl, 0.05 M ascorbic acid, 0.1 mM dopamine, 0.1 mM uric acid, 0.1 mM fructose, and 0.1 mM lactic acid into 0.2 M NaOH at +0.8 V. (**c**) Calibration curves of lactate concentration versus response current at 0 °C, 26 °C, 36 °C, and 100 °C, demonstrating stable detection across the full temperature range. (**d**) Temperature-dependent sensitivity variation from 0 to 100 °C, with error bars representing standard deviation from triplicate measurements.

The practical applicability of the optimized GF/Au/Ni(OH)_2_ non-enzymatic lactate sensor was comprehensively evaluated through long-term stability, anti-interference capability, and wide-temperature adaptability assessments. The long-term stability of the GF/Au/Ni(OH)_2_ sensor was evaluated by measuring its amperometric response to 0.1 mM lactate over a period of 14 days. Between measurements, the sensors were stored at ambient temperature (25 °C) in a desiccator under vacuum. Prior to each test, the electrodes were rehydrated by immersion in 0.2 M NaOH for 10 min to ensure restoration of the Ni(OH)_2_/NiOOH active layer. Figure 6a presents the continuous tracking of the amperometric response to 3 mM lactate over a 14-day storage period. The sensor demonstrates exceptional operational stability, with the initial response current retaining 99.3% on day 3, 98.6% on day 7, and 98.1% on day 10. After 14 days of continuous testing, the sensor still maintains 97.8% of its initial response current, corresponding to a performance degradation rate of merely 2.2%. This outstanding long-term durability is attributed to the robust inorganic catalytic architecture of the GF/Au/Ni(OH)_2_ composite, which circumvents the intrinsic instability issues associated with enzymatic sensors, such as protein denaturation and enzyme deactivation under ambient storage conditions. The selectivity of the sensor was evaluated by monitoring its amperometric response to common electroactive interferents that typically coexist with lactate in complex biological matrices. To impart selectivity to the sensor, a Nafion outer membrane was introduced onto the GF/Au/Ni(OH)_2_ electrode surface. Specifically, after Ni(OH)_2_ electrodeposition, 10 μL of 1 wt% Nafion ethanol solution was drop-casted onto the electrode active area and dried at room temperature for 30 min. The sulfonate groups (–SO_3_^−^) of Nafion are negatively charged in aqueous alkaline solution, enabling electrostatic repulsion of anionic interferents such as ascorbic acid (AA) and uric acid (UA), while allowing neutral/weakly charged lactate molecules to permeate through and reach the underlying Ni(OH)_2_/NiOOH catalytic layer. As depicted in Figure 6b, the interference assessment involved sequential addition of 0.05 mM NaCl, 0.5 mM ascorbic acid, 0.1 mM dopamine, 0.1 mM uric acid, and 0.1 mM fructose into a 0.2 M NaOH solution, followed by the introduction of 0.1 mM lactic acid. The sensor exhibits negligible current changes upon addition of the interfering species, whereas a significant and immediate current response is observed upon lactate addition. The minimal interference from NaCl confirms the excellent ionic stability of the sensor in high-salinity environments, while the resistance to ascorbic acid, dopamine, uric acid, and fructose—species that are readily oxidizable at conventional electrode surfaces—demonstrates that the combined Nafion barrier/Ni(OH)_2_/NiOOH catalytic system achieves high selectivity toward lactate oxidation. These results confirm that the GF/Au/Ni(OH)_2_/Nafion non-enzymatic lactate sensor possesses excellent selectivity for lactate, meeting the stringent requirements for high-precision analysis of complex biological samples.

Furthermore, to comprehensively assess the applicability of the sensor under challenging operational environments, its detection performance across an exceptionally broad temperature range was examined. Figure 6c displays the calibration curves of lactate concentration versus response current at 0 °C (low temperature), 26 °C (room temperature), 36 °C (near-physiological temperature), and 100 °C (extreme high temperature). The experimental results indicate that the GF/Au/Ni(OH)_2_ non-enzymatic lactate sensor exhibits remarkable temperature tolerance, maintaining stable and efficient detection performance across the entire 0–100 °C range. All four temperature conditions yield well-defined linear responses with comparable sensitivity trends, demonstrating that the inorganic catalytic mechanism is fundamentally independent of thermal fluctuations that would typically denature biological enzymes. Figure 6d quantifies the temperature-dependent sensitivity variation, with error bars representing the standard deviation from triplicate measurements. The sensitivity increases progressively with temperature, rising from approximately 1030 μA mM^−1^ cm^−2^ at 0 °C to 1120 μA mM^−1^ cm^−2^ at 26 °C, 1230 μA mM^−1^ cm^−2^ at 36 °C, and reaching 1290 μA mM^−1^ cm^−2^ at 100 °C. This positive temperature coefficient is consistent with the thermally activated nature of electrocatalytic oxidation kinetics, where elevated temperatures enhance the diffusion coefficient of lactate molecules and accelerate the charge transfer rate at the Ni(OH)_2_/NiOOH active sites. This exceptional thermal versatility establishes an important foundation for the practical deployment of the sensor in diverse demanding scenarios. In industrial bioprocessing and food quality control, the sensor enables accurate lactate monitoring during fermentation processes that may operate at sub-ambient or elevated temperatures, as well as during cold-chain logistics where real-time tracking of metabolic activity is essential. In clinical diagnostics and sports physiology, the sensor maintains reliable performance at physiological temperatures (36–40 °C) while tolerating thermal excursions during device operation or environmental exposure. The robust temperature characteristics ensure that the sensor can adapt to various harsh environments without compromising detection accuracy, from low-temperature transportation to industrial processing conditions. Collectively, the combination of outstanding long-term stability, superior anti-interference capability, and unprecedented wide-temperature operational range validates the viability of the GF/Au/Ni(OH)_2_ composite electrode for advanced applications in next-generation wearable health-monitoring systems and industrial on-site analytical platforms.

A comparative performance summary (Table 1) contrasts the analytical parameters of our GF/Au/Ni(OH)_2_ platform with literature-documented enzyme-free electrochemical lactate sensors, revealing marked superiority in both detection limit and sensitivity. The superior electrochemical performance of this sensor originated from the outstanding electrical properties of graphene fibers as substrate electrodes and the bimetallic layer structure formed by Au particles and Ni(OH)_2_ nanoparticles. The exceptional conductivity and high specific surface area of graphene fibers allowed a large amount of nanocatalytic materials to be anchored onto the fiber surface. Sequential electrodeposition of Au and Ni(OH)_2_ onto graphene fibers enhanced electron transport between lactate oxidation sites and the conductive scaffold. Furthermore, the dual-component Au/Ni(OH)_2_ coating created numerous active sites for lactate oxidation, substantially improving both the detection limit and sensitivity.

## 4. Conclusions

In summary, this investigation introduces a non-enzymatic lactate sensor built upon GF substrates, where a GF/Au/Ni(OH)_2_ hybrid electrode was assembled via sequential electrodeposition. Systematic optimization of deposition conditions elucidated a quantitative relationship between surface morphology and electrochemical behavior, exposing a fundamental compromise between the abundance of active sites and charge transfer efficiency. Peak performance was realized when both Au and Ni(OH)_2_ were deposited for 900 s. At this optimal condition, the interconnected Au nanostructures establish an efficient electron transport network, while the hierarchically wrinkled surface ensures unimpeded mass transport, achieving a synergistic balance between conductive network density and active site accessibility. The optimized sensor afforded a sensitivity of 1.24 mA mM^−1^ cm^−2^ and an unprecedentedly broad temperature window of 0–100 °C. This exceptional thermal versatility, enabled by the robust inorganic catalytic architecture, ensures stable operation across a broad temperature range. The device further exhibited outstanding reproducibility, superior interference tolerance, and extended durability. This research delivers a logical design framework for balancing catalytic activity with transport properties in MMO composites, establishing a dependable foundation for advanced applications in next-generation wearable health-monitoring systems.

## Figures and Tables

**Table 1:** Performance comparison of non-enzymatic lactate sensors.

Non-Enzymatic Lactate Sensor	Sensitivity (μA·mM^−1^·cm^−2^)	Limit of Detection (μM)	Ref
Au/PDA/PtMN	11.25	50	[14]
NiO	24	8.1	[10]
Ni(OH)_2_	12.09	70	[10]
(NiO)NPs	62.35	27	[58]
AuR-DTSP-LOx	1.49	100	[59]
HS-NiS	0.65	0.5	[23]
NADH/LDH/CeO_2_	8400	200	[60]
Ni(OH)_2_-ITO	132.50	6.7	[24]
Ni(OH)_2_NP/p-SPE	0.036	500	[61]
NiOOH/Ni(OH)_2_	80.00	0.15	[25]
NiS-NC@NiS-MS	2.20	0.5	[62]
GF/Au/Ni(OH)_2_	1239.30	1.82	This work

## Data Availability

The data presented in this study are available on request from the corresponding author due to research confidentiality restrictions.
